# MetaRanker 2.0: a web server for prioritization of genetic variation data

**DOI:** 10.1093/nar/gkt387

**Published:** 2013-05-22

**Authors:** Tune H. Pers, Piotr Dworzyński, Cecilia Engel Thomas, Kasper Lage, Søren Brunak

**Affiliations:** ^1^Department of Systems Biology, Center for Biological Sequence Analysis, Technical University of Denmark, Lyngby, Denmark, ^2^Division of Endocrinology and Center for Basic and Translational Obesity Research, Children’s Hospital Boston, USA, ^3^Broad Institute of the Massachusetts Institute of Technology and Harvard, Cambridge, MA, USA, ^4^NNF Center for Protein Research, Faculty of Health Sciences, University of Copenhagen, Copenhagen, Denmark, ^5^Pediatric Surgical Research Laboratories, Massachusetts General Hospital, Boston, MA, USA and ^6^Harvard Medical School, Boston, USA

## Abstract

MetaRanker 2.0 is a web server for prioritization of common and rare frequency genetic variation data. Based on heterogeneous data sets including genetic association data, protein–protein interactions, large-scale text-mining data, copy number variation data and gene expression experiments, MetaRanker 2.0 prioritizes the protein-coding part of the human genome to shortlist candidate genes for targeted follow-up studies. MetaRanker 2.0 is made freely available at www.cbs.dtu.dk/services/MetaRanker-2.0.

## INTRODUCTION

Genetic association studies provide near-unbiased screens of common and rare variants’ association with complex traits. Genome-wide association (GWA) studies highlight distinct loci, and thereby reduced, yet sizable, sets of genes among which to search for likely causal candidates (1). Complex trait-based exome chip analyses (2) and exome sequencing studies (3) highlight coding mutations within specific genes, but generally lack statistical power to establish significant associations. Therefore, association studies and rare variant analyses typically rely on downstream bioinformatics analysis, to further reduce their shortlisted candidate genes to numbers that allow in depth experimental follow-up studies.

Genetic alterations may trigger a downstream cascade of changes in cellular states (4). Consequently, analyses of genetic variation data have been augmented by integration with complementary data sets, among others differential- or tissue-specific gene expression data (5), protein–protein interaction data (6) or existing literature-based knowledge (7). Although there are highly specialized tools that facilitate gene prioritization in chromosomal regions [e.g. Endeavour (8), or Prioritizer (9)], or GWA loci [e.g. GRAIL (10), or DAPPLE (11)], there is only a limited number of tools that allow researchers to combine their in-house portfolio of genomics data sets with relevant publicly available data sets [see (12) for an in-depth review of existing gene prioritization methods]. One of these approaches is MetaRanker 1.0 (13), our previously published approach, which augments genetic analyses by prioritizing the genome in relation to a specific phenotype of interest through integration of heterogeneous and complementary data sources. MetaRanker facilitates integration of the following data types:

- Single nucleotide polymorphism (SNP) to phenotype associations from GWA studies, which represent a rapidly growing resource of unbiased common variant associations.
- High-confidence protein–protein interaction networks centred on proteins encoded by user-defined phenotype-related susceptibility genes, which may contribute with non-obvious pathway-based information.
- Data from linkage studies capturing co-segregation of chromosomal regions and disease-specific phenotypes, thereby highlighting chromosomal intervals likely to harbour causal genes.
- Quantitative data on disease similarities, which may add information that exploit overlaps in disease definitions.
- Tissue-specific or differential gene expression data from microarray or sequencing-based studies.

These data sources are treated as evidence layers that can be used in any combination, and are collapsed into an integrative meta-layer. We validated MetaRanker 1.0 by discovering a novel bipolar disorder susceptibility locus (rs1049583, near *YWHAH*), which we replicated through genotyping in independent cohorts. Another tool that allows prioritization of disease genes by integration through various data types is CANDID (14). We benchmarked MetaRanker successfully against this method.

In this article, we describe MetaRanker 2.0, which extends our original approach in several significant ways:

- Integration of new user-specified data sets, such as data from next-generation sequencing studies, or additional gene expression experiments. (User input: Gene IDs and gene-based scores).
- Integration of copy-number variation data. (User input: Chromosomal regions),
- Improved gene ranking based on large-scale text-mining. (User input: Key words).
- Improved GWA data-based scoring of genes.
- Improved usability of the web server.

## MATERIALS AND METHODS

**Figure 1.**
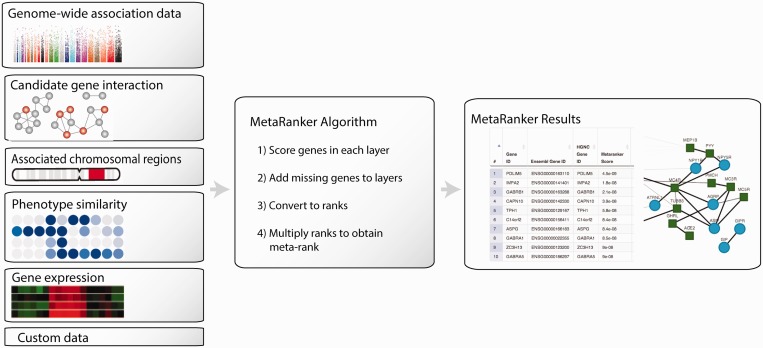
Overview of MetaRanker 2.0 workflow. The user submits one or several types of data sets (evidence layers), which subsequently are converted into ranks and integrated to yield a prioritized meta-rank. Genes likely to be associated with the trait—given the evidence layers—will be ranked at the top of the meta-rank.

Below the MetaRanker 2.0 improvements are briefly described. Please refer to Pers *et al.* (13) and Figure 1 for a description of the original algorithm and data sets used by MetaRanker.

### Integration of user-specified data sets

MetaRanker 2.0 allows the user to upload lists of genes and their scores. Scores can denote tissue-specific expression levels, binary values indicating causality, *P*-values from gene-based associations tests, or any other type of gene-based score. The web server supports upload of several different gene nomenclatures (Ensembl gene IDs, Hugo gene symbols, and Entrez gene IDs), and up to five custom evidence layers.

### Integration of copy-number variation data

MetaRanker 1.0 facilitated integration of linkage data by requiring the user to upload chromosomal bands. Large-scale genotyping based on high-density SNP arrays, microarray-based comparative genomic hybridization and sequencing have superseded traditional linkage analysis. MetaRanker 2.0 allows upload of chromosomal regions based on physical coordinates (e.g. chr4:300,123-404,567) to facilitate both linkage data and copy number variation data. Genes overlapping with these user-specified regions are collectively weighted higher than the rest of the genes in the human genome.

### Improved gene ranking based on large-scale text-mining

The text-mining layer in MetaRanker 2.0 quantifies the association between genes and phenotypes based on 11 620 324 abstracts contained in the PubMed database MEDLINE (download date 22 November 2011). We have constructed word vectors for 18 804 genes, and 15 807 phenotypic Medical Subject Headings (MeSH) terms. Using an approach that resembles the methodology applied by GRAIL (10), and previous work by us (15), we first normalize word vectors to adjust for publication biases, and then use the cosine angle between term vectors to compute pairwise similarities between genes and MeSH terms. In this new framework, the user can rank genes based on combinations of phenotypic terms by using logical AND, and OR relationships. Compared with the MetaRanker 1.0 disease similarity layer, which was based on text-mining of the Genecards database (16), the new layer provides a more flexible and extensive approach to literature-based gene ranking.

### Improved GWA data-based scoring of genes

MetaRanker 1.0 scored genes by (i) mapping SNPs to genes (based on physical proximity), (ii) assigning *P*-values to genes based on the best-associated SNP and (iii) adjusting gene *P*-values by the number of independent SNPs mapped to the given gene (a correlate for gene length). A recent paper (17) has proposed that in situations where many SNPs map to the same gene (typically observed in GWA studies), a method developed by Li and Ji (18) performs superior compared with the independent number of SNPs calculation algorithm proposed by Galwey (19) and implemented in MetaRanker 1.0. Therefore, we implemented the Li and Ji correction method in MetaRanker 2.0.

### Improved usability

We have improved the usability of MetaRanker by implementing several key features: First, in the new web server, we have eased user handling by enabling several analyses simultaneously. Second, since GWA study evidence layer-based analysis, and/or the text-mining layer-based analyses, take dozens of minutes to complete, we have added progress bars for each layer that was included in the analysis. Third, we have added searching, and column-specific sorting of results. Finally, we have added interactive visualization that displays the 20 best-associated genes, along with their high-confidence protein–protein interaction partners. Interaction data originates from the InWeb database (15), and the user can interactively explore the network by re-orienting nodes and edges.

## EXAMPLES ON METARANKER 2.0 ANALYSES

MetaRanker 2.0 represents a versatile tool that facilitates integration of several data types. Below, we briefly illustrate three ways MetaRanker 2.0 can be used to prioritize genes for follow-up studies.

### Prioritization of genes based on GWA data

MetaRanker 2.0 facilitates prioritization of genes based on user-specified GWA summary statistics. Summary statistics can be uploaded as text or compressed files (.zip, .tar or .gz file formats). The user can add any other combination of the evidence layers. The results consist of a ranked list of prioritized genes, along with information on the number of SNPs mapped to each gene, each gene’s best-associated SNP and the number of independent tests per gene.

### Prioritization of genes based on rare-variant analyses data

Single-marker analyses of rare-variant data often have sub-optimal power to detect statistically significant associations (20). MetaRanker 2.0 facilitates prioritization of genes based on user-specified rare-variant association data (e.g. from exome chip analyses or exome sequencing studies) by integrating gene scores [e.g. sequence kernel association test *P*-values (21)] with any other combination of evidence layers. This can be accomplished by uploading gene-based scores to a custom layer, and, depending of the type of gene score, enabling either ascending or descending sorting (*P*-values, for instance, should be sorted in ascending order).

### Prioritization of genes based on protein–protein interaction-based guilt by association scoring

MetaRanker 2.0 is not limited to analyses of GWA data, or data from exome chip or exome sequencing studies. The web server can also be used to rank genes based on their gene products’ propensity to physically interact with a user-specified set of gene products. Examples on user-specified gene sets are phenotypic gene sets from the Online Mendelian Inheritance in Man database (22), or genes that upon knock-out in model organisms resemble the phenotype for the trait under investigation [e.g. genes from the Mouse Genome Database (23)].

## BENCHMARKS

In our original article, we successfully used genotyping of independent bipolar disorder cohorts to show that MetaRanker 1.0 enabled prediction of likely causal disease genes. In addition, we showed that MetaRanker 1.0 performed superior to CANDID in benchmark studies of type 2 diabetes and bipolar disorder. In this work, we conducted additional benchmarks and show that MetaRanker 2.0 enriches for causal human stature genes. We report Receiver Operating Characteristic (ROC) curves (24) and Area Under the Curve (AUC) estimates for MetaRanker 1.0, MetaRanker 2.0, and CANDID.

Human stature is for several reasons well suited as a benchmark: (i) well-powered GWA data are available (25), (ii) many genes with Mendelian variants that are known to cause either overgrowth or small stature have been identified (25), (iii) gene expression data from rodent growth plates, a highly relevant tissue in relation to human stature, was recently published, (iv) knock-out data from mice, phenotyped for well-defined skeletal phenotypes, is available, and (v) it has previously been shown that many height genes are well-recorded in the literature (10). We constructed human stature-specific evidence layers as shortly outlined below. (All data sets can be downloaded from www.cbs.dtu.dk/services/MetaRanker-2.0.)

### MetaRanker 2.0 data sets

We downloaded the summary statistics from the well-powered Lango-Allen *et al.* human height GWA study that was based on 183 727 genotyped individuals (http://www.broadinstitute.org/collaboration/giant/index.php/GIANT_consortium_data_files) (25), and uploaded them to the MetaRanker 2.0 GWA data layer. As input to the MetaRanker 2.0 protein–protein interaction layer, we used a list of 241 genes, which in the OMIM database have been reported to be causal to skeletal growth disorders [the list was provided in Lango-Allen *et al.* Supplementary Table 10 (25) and was compiled independently from the GWA study results]. As input to the MetaRanker 2.0 text-mining layer, we used the terms ‘body height’ and ‘growth disorders’ and enabled the logical ‘OR’ relationship to rank genes higher if they were co-mentioned with one or the other of these two terms. Finally, we retrieved 1002 human homologue genes from the Mouse Genome Database that upon knock-out resulted in skeletal growth-related phenotypes, and uploaded them to the MetaRanker 2.0 custom layer enabling the option that all other genes in the human genome should be scored worse than these genes.

### CANDID data sets and parameterization

For the literature layer, we used the same input terms as used in the MetaRanker 2.0 analysis. For the association layer, we uploaded the Lango-Allen *et al.* GWA study SNPs and applied the commonly used genome-wide significance threshold of *P* < 5 × 10^−^^8^ as the cut-off because the SNP count exceeded the number of SNPs supported by CANDID. We included the ‘Interactions’ layer, and for the custom layer, we uploaded the same Mouse Genome Database gene set as described above. All layers were weighed equally, and non-protein coding genes were excluded.

### Benchmark

**Figure 2.**
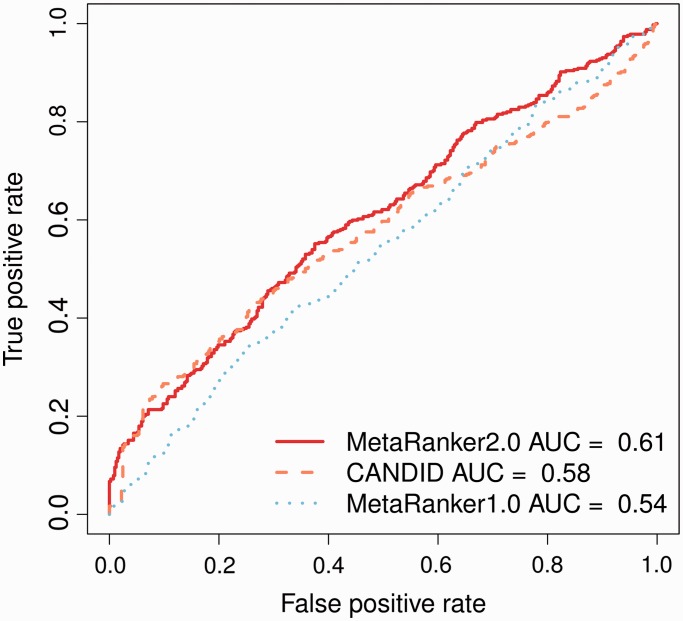
ROC curves and AUC estimates for MetaRanker 2.0, MetaRanker 1.0 and CANDID. MetaRanker 2.0 performs superior to both MetaRanker 1.0 and CANDID, as illustrated by the higher AUC obtained by MetaRanker 2.0.

As positive genes we used a recently published list of 408 genes differentially expressed in rodent growth plate experiments (26). Note, that this data set was published after the download date of all other data sets used in this analysis. As negative genes, we used a random sample of 408 genes, and ensured that none of them overlapped with any of the OMIM and Mouse Genome Database genes. For ROC curve constructions and AUC calculations, we confined ourselves to genes scored in both approaches, and used the rank of positive and negative genes as scores. We found that MetaRanker 2.0 performed superior to MetaRanker 1.0, and CANDID (Figure 2). AUCs were 0.61, 0.54 and 0.58, respectively, and true positive rates at the 5% cut-off were 0.05, 0.03 and 0.05, respectively. Compared with CANDID, MetaRanker 2.0 permits the user to upload a larger number of phenotype-specific data sets—an important advantage that might have resulted in the increased performance. The increasing availability of predictive biological data might further increase the advantage of the MetaRanker 2.0 approach compared with other approaches that do not allow the user to upload his own data sets.

## CONCLUSIONS

We show that MetaRanker 2.0 provides an easy to use and flexible platform for gene prioritization based on integration of multiple heterogeneous data sets. We successfully benchmarked our tool against CANDID, another tool for multiple-evidence genomic data integration.

## FUNDING

Danish Council for Independent Research Medical Sciences (FSS) (to T.H.P.); Novo Nordisk Foundation (to S.B.). Funding for open access charge: Center for Biological Sequence Analysis, Department of Systems Biology, Technical University of Denmark.

*Conflict of interest statement*. None declared.
